# Methodological identification of anomalies episodes in ECG streams: a systematic mapping study

**DOI:** 10.1186/s12874-024-02251-0

**Published:** 2024-06-04

**Authors:** Uzair Iqbal, Riyad Almakki, Muhammad Usman, Abdullah Altameem, Mubarak Albathan, Abdul Khader Jilani

**Affiliations:** 1 Department of Artificial Intelligence and Data Science, National University of Computer and Emerging Sciences, Islamabad, Pakistan; 2 Information Systems Department, College of Computer and Information Sciences, Imam Mohammad Ibn Saud Islamic University (IMSIU), 11432 Riyadh, Saudi Arabia; 3 Department of Computer Science and Technology, Harbin Institue of Technology, Harbin, Heilongjiang, China; 4 Information Systems Department, College of Computer and Information Sciences, Imam Mohammad Ibn Saud Islamic University (IMSIU), Riyadh, Saudi Arabia

**Keywords:** Myocardial infraction, Systematic literature review, Classification, T wave, And Electrocardiography

## Abstract

An electrocardiogram is a medical examination tool for measuring different patterns of heart blood flow circle either in the form of usual or non-invasive patterns. These patterns are useful for the identification of morbidity condition of the heart especially in certain conditions of heart abnormality and arrhythmia**.** Myocardial infarction (MI) is one of them that happened due to sudden blockage of blood by the cause of malfunction of heart. In electrocardiography (ECG) intensity of MI is highlighted on the basis of unusual patterns of T wave changes. Various studies have contributed for MI through T wave’s classification, but more to the point of T wave has always attracted the ECG researchers. *Methodology.* This Study is primarily designed for proposing the combination of latest methods that are worked for the solutions of pre-defined research questions. Such solutions are designed in the form of *the* systematic review process (SLR) by following the Kitchen ham guidance. The literature survey is a two phase’s process, at first phase collect the articles that were published in IEEE Xplore, Scopus, science direct and Springer from 2008 to 2023. It consist of steps; the first level is executed by filtrating the articles on the basis of keyword phase of title and abstract filter. Similarly, at two level the manuscripts are scanned through filter of eligibility criteria of articles selection. The last level belongs to the quality assessment of articles, in such level articles are rectified through evaluation of domain experts. *Results.* Finally, the selected articles are addressed with research questions and briefly discuss these selected state-of-the-art methods that are worked for the T wave classification. These address units behave as solutions to research problems that are highlighted in the form of research questions. *Conclusion and future directions.* During the survey process for these solutions, we got some critical observations in the form of gaps that reflected the other directions for researchers. In which feature engineering, different dependencies of ECG features and dimensional reduction of ECG for the better ECG analysis are reflection of future directions.

**Supplementary Information:**

The online version contains supplementary material available at 10.1186/s12874-024-02251-0.

## Introduction and Background

Pattern recognition is a game changer in time series data. Differentiating between regular and irregular patterns is highly desirable in such cases that involve urgency and risk factors. In the context of these statements, data from MRI monitors the unusual behavior of the brain (brain tumor), data from CT SCAN relates to the broader picture of the human body, and data from ECG highlights the usual and non-invasive heart activities. The scope of this article deals with anomalous behaviors of the heart in terms of ECG analysis. Unusual activities of the heart are referred to as anomalous behavior that represents cardiovascular diseases (CVDs). Accurate and robust classification of these CVDs has especially attracted cardiologists and ECG researchers. Some of the CVDs are critical for diagnostic purposes like one of them is a myocardial infarction (MI). MI depends on the effects in the ST part and T wave and main focus is on changes of the T wave. The primary cause of myocardial infarction (MI) stems from anomalous T-wave episodes [1, 2]. Automated classification of T-wave episodes is a game changer for cardiologists and ECG researchers.

Accurate classification of T wave demonstrates the concept of different types of T wave episodes like flattened T wave, inversion T wave, and negative T wave. Identification of the exact characteristics of flattened T is an open research problem for ECG researchers. In operational investigations of literature for better classification of T-wave episodes, In one case, the identification of flattened T-wave characteristics involves assessing parameters such as time duration, peak value, and start and end times. Additionally, dependencies on various features related to T-wave episodes, such as the utilization of the R peak as a reference point for calculating the T-onset parameter [3], play a significant role in classifying diverse T-wave episodes. These calculations of dependency factors are crucial for achieving accurate and robust classification of different T-wave episodes, highlighting the importance of the literature problem definition. During the literature survey, numerous studies directly and indirectly related to T-wave classification were discovered, underscoring the relevance of this research. In several studies, one of them is a Manifold algorithm that works for anomaly detection in ECG data and gets the accuracy at 96% level. Such an algorithm works in three phases. In the first phase, segmentation, and feature extraction are performed. In the second phase, the manifold structure was discovered and mapped with findings. The last phase deals with the anomaly detection and recognition [2, 3]. Similarly, the Myocardial infraction detection algorithm MI detection algorithm surpasses the accuracy of traditional algorithms, achieving an impressive 98% accuracy rate [4]. It has been specifically designed for the detection of abnormalities in the ST segment and T-wave [5–11]. The MI detection algorithm is particularly focused on distinguishing between ST-segment elevation (STEMI) and non-ST segment elevation (NSTEMI) cases [12–16]. This study is intended to enhance the accuracy of state-of-the-art methods in classifying various T-wave episodes.

This article is designed for solutions to the defined problems through a literature survey. Such a solution belongs to the robust and accurate classification of different T wave episodes by using the hybrid approach. Concepts of behavior analytics are integrated with parametric analysis of the T wave for calculating the dependencies factor. This factor plays a critical role for another perspective in terms of classifying the T wave anomalies, such perspective reflects in terms of different neural models [17–19]. These three different perspectives are merged for a better and more accurate classification solution [20–22].For the conduction of this hybrid approach, the dependencies indicator of T wave parameters is a central theme of this approach like interconnectivity between T-onset, T-offset, T peak value, and T wave time duration [23–29].

By using our best knowledge this domain, so far, we are the first ones who proposed the best possible solution in terms of accurate and efficient classification of different T wave episodes based on nature. Such natural identification entirely relies on the parameters of T wave. In literature, a number of studies found that build a reasonable accuracy level in terms of classification of different T wave episodes, but due to these methods, the difference between flattened T wave and inversion T wave is still unclear [30–34]. Accurate and robust classification of different T wave episodes relies entirely on the identification of the values of T wave parameters especially in terms of T-onset and T-offset [35–41]. Such findings are further helpful for highlighting the intensity of MI in terms of ST-segment elevation and ST-segment depression [42–46].

This study delivers the good practice of Kitchenham guidelines [47]. Execution of this guideline in a systematic way like the first phase reflects the collection of 97 articles through four primary databases. These 97 studies were rectified by applying three different rectification levels and after these levels finally fetched the 26 articles. These 26 studies are the primary source for the execution of this review article. The core focus of this systematic study is to review the latest techniques of T wave anomaly classification and detection. A summary of our contributions is highlighted below.

1. Execution of in-depth classification based on parametric analysis of different T-wave episodes.
2. Introducing the discussion session of dependencies factors that work for robust and better classification.
3. Proposed three-way perspectives with the help of selected articles. These three-way perspectives are designed for robust and accurate classification by using state-of-the-art terminologies like parametric analysis of T waves and classification of different T waves through neural models.

The rest of the paper is organized into different sections. Sect. " Selection Protocols" will deliver the complete methodology of research conduction. Section 3 covers the discussion portion of selected studies. Sect. " Methodological Comparison" delivers a short summary of relevant articles in the sense of results. Sects. " Taxonomical View:Three- Way Handshake" and 6 highlight the taxonomy and the future directions. Section 7 concludes the summary of this article.

## Selection protocols

Healthcare systems give special consideration to scenarios involving risk factors, particularly in the context of cardiac process monitoring. Even minor details of heart activities cannot be overlooked in this process. Accurate and timely identification is crucial for diagnosing various cardiac conditions. In light of these considerations, providing specific attention to diagnostic purposes is essential when dealing with patients affected by myocardial infarction. In myocardial infarction, the most significant part is to identify the defective shape of the T wave. Classification is used to identify the abnormalities of T wave but identifying the abnormality of Flatten T wave is still a problem for researchers and cardiologists. This article delivers a way how to sort out the robust and accurate classification of the different T-wave episodes by manipulating the literature [48, 49].

### Research questions

- RQ1: how it is possible to classify the different anomalies of T wave through parameters of T wave?
- RQ2: Which Parameters of T wave plays a vital role for identification the different T wave nature?
- RQ3: how neural models are the best methods for identifying the feature dependencies of ECG?
- RQ4: What are the main factors that play a critical role in the classification of different T-wave episodes?

### Keywords for searching

The formation of SLR is built on the base of keywords, and such keywords are used in the query string for the collection of articles. Table 1 highlights the possible synonyms in this SLR. Table 2 reflects the possible acronyms.

**Table 1:** Highlight the keywords with synonyms

Keywords	Synonyms
T-onset	Start time of T wave
T-offset	End time of T wave
T wave Peak value	Amplitude of T wave
Inverted T wave	Inversion T wave
T wave slope	Deflection value of T wave
Anomaly detection	irregular pattern detection
Neural Network	Artificial Neural Network
Backpropagation Algorithm	Backpropagation Approach
Early detection of T-wave	In time detection of T wave

**Table 2:** Highlight the keywords with Acronyms

Keywords	Acronyms
Electrocardiography	ECG
Cardiovascular Diseases	CVDs
Myocardial infarction	MI
ST-Segment Elevation myocardial infarction	STEMI
Non-ST Segment Elevation myocardial infarction	NSTEMI
Backpropagation	BP
Neural Network	NN
Artificial Neural Network	ANN
Behavior Analytics	BA
Ontology Behavior model	OnToB model
Multi-Resolution Analysis	MRA
Support Vector Machine	SVM
Convolution neural network	CNN
Recurrent neural network	RNN
k nearest neighbor neural network	KNN
Probabilistic neural network	PNN

### Data source

Four primary databases that are highlighted in Table 3 are used for extraction articles.. This extraction process is executed by considering the last twenty years of articles

**Table 3:** Electronic databases

Identifier	Databases	URL
*DB1*	IEEE Xplore	http://ieeexplore.ieee.org
*DB2*	Springer Link	http://link.springer.com
*DB3*	Science Direct	http://www.sciencedirect.com
*DB4*	Scopus	https://www.scopus.com

The following items are searched based on research questions and relevant literature. Proper classification of the various T wave anomalies based on nature is a hot issue for medical professionals especially flattened T wave. Accurate and robust classification of different T wave episodes is one of the primary purposes of this SLR. For this purpose, the first stage is to discuss all the possible interconnect dependencies of T wave parameters. This SLR is a joint alignment of different behaviors of different T wave episodes along with a discussion of T wave dependencies analysis. Finally, the usage of the above combination with neural models works for the achievement of robust and accurate classification of different T waves [50–54]. In the context of accurate and robust classification, the proposed idea is executed with the query string generation method. Such a method is stated below.

- (1). “Behavior impact analysis” OR “Negative Behavior sequences” OR “Behavior Informatics.”
- (2). “OnTo Behavioral Model” OR “Behavior Checkpoint”.
- (3). “Neural networks” OR “Different methods of neural models for classification” OR “ECG classification through neural models.”
- (4). “ECG morphology analysis” OR “ECG feature extraction” OR “ECG feature analysis.”
- (5). “T wave behavior” OR “T wave start time” OR “T-onset.”
- (6). “T wave behavior” OR “T wave end time” OR “T-offset.”
- (7). “T wave behavior” OR “T wave peak value” OR ‘T wave amplitude” OR.
- (8). “T wave behavior” OR “T wave time duration.”

These above search items are combined in the form of a string generator by using the Conjunction (AND) and Disjunction (OR) operators. The result of a search string for the collection of relevant literature is as follows:

(“Behavior impact analysis” OR “Negative Behavior sequences” OR “Behavior Informatics” OR” OnTo Behavioral Model” OR “Behavior Checkpoint”) AND (“Neural networks” OR “Different methods of Neural models for Classification” OR “ ECG classification through neural models”) AND (“ECG morphology analysis” OR “ECG feature extraction” OR “ECG feature analysis”) AND( “T wave behavior” OR “T wave start time” OR “T-onset”) AND( “T wave end time” OR “T-offset” OR “T wave behavior”) AND(“T wave behavior” OR” T wave peak value” OR ‘T wave amplitude”) AND (“T wave behavior” OR “T wave time duration”).

### Inclusion and Exclusion Criteria

The above connection string is valuable for the selection of related articles. These articles passed through the other filtration process that is mentioned in Table 4. The formation of inclusion and exclusion checks in Table 4 is formated based on technical-oriented clinical research contributions which belongs to human participants for the collection of data and execution of experiments with ethical clearance.^1^. The schema of inclusion and exclusion criteria relies on the above connection string. At the first phase, the inclusion of articles primarily depends on any novel approach or technique for the detection of anomalies of T wave. For understanding the different anomalous behaviors of the T wave in ECG, the induction of behavior analytics articles delivers the better knowledge of different dependencies concept. Conversely, articles excluded on the basis of unclear results and invalid techniques. Such type of articles is completely aborted. A few of the traditional techniques are also excluded due to their least effectiveness in percentage accuracy measurement..

**Table 4:** Inclusion and exclusion criteria

Schema of Inclusion	
IS1	Research Articles that peer-reviewed
IS2	Articles introducing any novel approach for finding the solution of anomalies in ECG morphology
IS3	Articles highlighted anomalies classification on the basis of T wave Parameters
IS4	Inclusion behavior analytics techniques in real-time system which are suitable for anomalies detection of T wave
IS5	Result oriented techniques be a part of literature
Schema of Exclusion	
ES1	Articles delivered unclear results or finding
ES2	Articles providing traditional techniques for classifier the anomalies due to lack of dependencies factor
ES3	Articles lacking validation introduce unverified techniques
ES4	Articles that are not written in the English

### Data extraction strategy

All the articles are selected after clearing the inclusion and exclusion stage. These research articles are assessed on the basis of parameters which are highlighted in Table 5. Specific information was extracted on the basis of predefined extraction items like Title, Publication venue,Publication Year,Approach (Feature analysis, Parametric discussion of T wave and classification of abnormalities in ECG through neural models),Research methods,Research questions addressed.

**Table 5:** Article extraction form

Data Source	Data Unit information
*Identifier*	S1
*Title*	ECG Patch Monitors for Assessment of Cardiac Rhythm Abnormalities
*Author*	S. Suave Lobodzinski
*Year of Publication*	2013
*Publication Type (Journal /Conference)*	Journal
*Publication Venue*	Progress in Cardiovascular Diseases
*Research Method (Investigational Approach/ SLR / A case study)*	Long-term monitoring investigational approach towards improvement of diagnostic yield
*Research Questions Address up*	*Directly: –––––––-*
	*Indirectly*: RQ4 satisfied, parametric discussion of ECG features

Above Table 5 displays the data extraction strategy in the form of first extracted identifier S1. Extraction policy is applied to all selected articles that captured unit information in tabularized form, and few samples are discussed in Appendix-A.

### Literature quality assessment

The quality assessment activity is the third stage of study selection flow that is highlighted in Table 5. At first stage of screening, the combination of both schema’s inclusion and exclusion are worked with assessment criteria of literature for the identification of relative research articles*.*

Queries of Table 6 are implemented on those articles that are selected after first rectification process. Answer the queries of Tables 6 in the format of YES, NO and PARTIALLY that is the next step for further improvement of rectification process by the induction of five domain reviews (Rev). These reviews mark the score of the second stage selected articles according to the format of 0.5,0 and 1(represents the PARTIALLY, NO, and YES).

**Table 6:** Assessment quality

Sr #	Assessment Questions	Answers		
		*Yes*	*No*	*Partially*
1	Research aims mapped with context of study			
2	Validation of novel based techniques maps with research designed			
3	Dependencies must be clearly stated			
4	Research objectives are clearly defined			
5	Behavior Analytics approach highlighted in the study			

### Selected Search Item

Execution of this systematic study relies on the rectification process, at first stage collection of 97 articles is done based on our best knowledge of the domain. In the next stage, another filter out of scope is worked on the basis of the title and abstract. After this stage, we get 60 articles out of 97 articles bank (excluded 28 articles on the basis of out of scope and 9 excluded on the basis of improper discussion towards problem). After exclusion of articles on such stage then again implement the next rectification process. At such point the articles based on the criteria for inclusion and exclusion that are highlighted in Table 4. In this rectification, we get 42 articles out of 60 articles (exclude 18 articles on the basis of another analysis discussion). Now after such processes again implement the second rectification process but this time consider such articles which are more in-depth discussion towards T wave anomalies, and also concern the quality parameters of literature that are mentioned in Table. The scoring range of 42 articles is displayed in appendix-B. Appendix-B displays the scoring calculator which works based on five reviewers (Rev). The selection of five reviewers on the bias of standard EASE 7 which covers the expertise, and potential computing interests of the reviewer considers the diversity and inclusion of reviewer^2^ After selection. After the Selection of reviewers mark the score based on the literature quality assessment. The range of scoring is 0.5 to 1, in this range 0 represents false assessment, 0.5 shows partially, and 1 indicates accurate response towards quality assessment.The next step in this rectification process is to set the low threshold value that is equal to 3. Such a statement indicates that when your article scores equal to or higher than the threshold point, then the inclusion of the article is possible otherwise skip that particular article. After this rectification process, we fetched 26 articles out of 42 (excluding 16 articles based on the disappointing result in terms of quality assessment). Figure 4 is a pictorial representation of the whole selection process of articles.

**Fig. 1:**
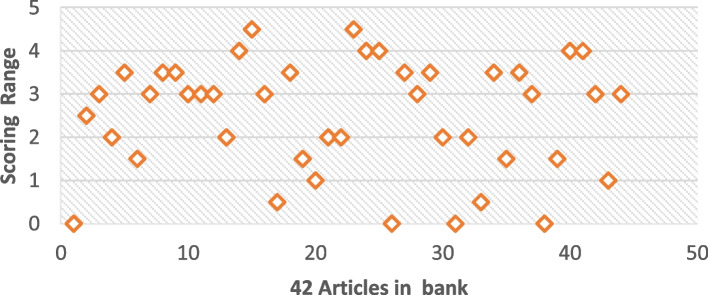
Articles maps with a scoring range

Execution of the scoring table at a stage of 42 articles reveals the importance of different reviewer thinking, such a thoughtful process is highlighted in Fig. 1. According to Fig. 1, the maximum range of scoring is 4.5, and the minimum one is 0.

**Fig. 2:**
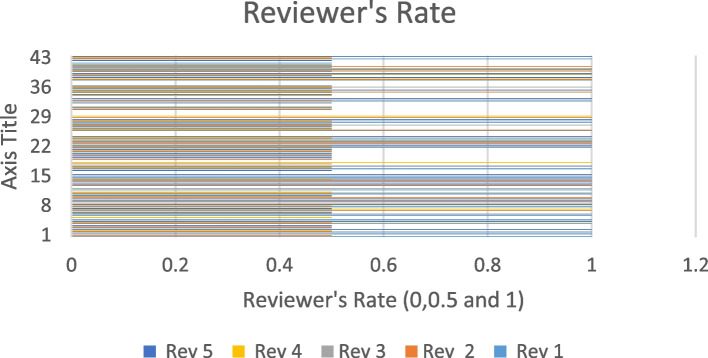
Rating ratio of different reviewers

In the scoring calculator, the 5 reviewers investigated each article and delivered the score with their best knowledge. The replica of the above statements is represented in the form of a showcase of reviewers’ trends in Fig. 2. The data mounted below showcase highlights the average, maximum, and minimum scoring rates. According to Fig. 2, the trend of the reviewer’s rating is displayed in such a manner that represents the article's rating in the form of 0. 0.5 and 1. Such a point indicates the selected 42 articles partially satisfied the pre-defined quality assessment, but with the help of this showcase, further rectification investigations are so simple.

**Fig. 3:**
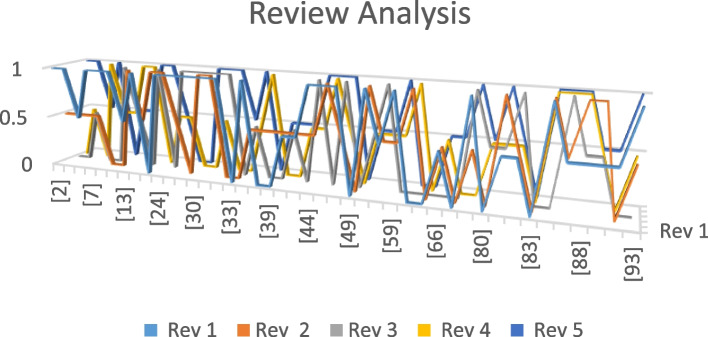
Reviewer analysis of selected articles

**Fig. 4:**
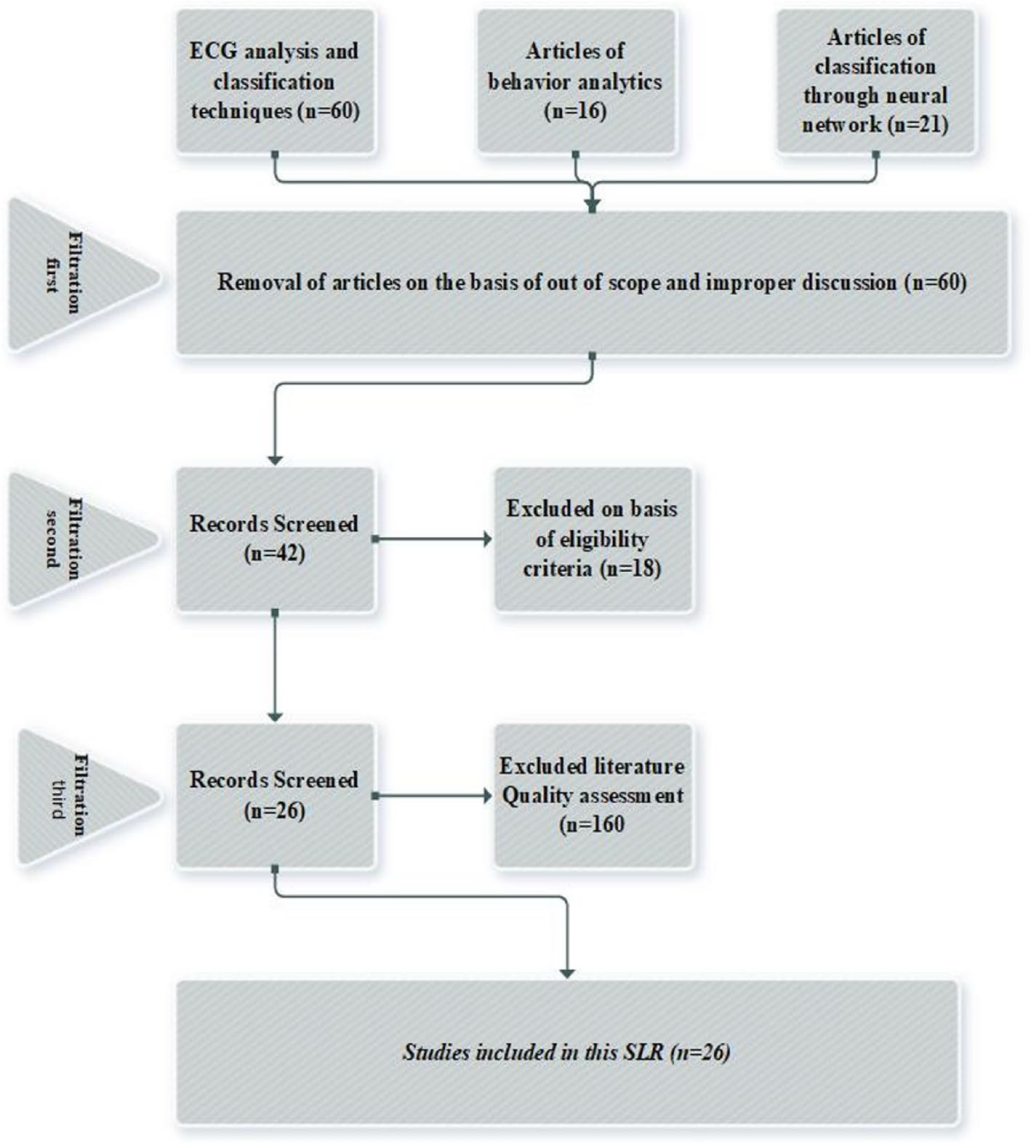
Selection Process of articles for composition of this systematic study

A further observation in terms of review analysis delivers a broad picture of selected articles that are mapped with a research problem. The expansion of the reviewer’s trend in the form of the reviewer’s analysis is represented in Fig. 3. Figure 3 represents the in-depth investigations that indicate the variations in the reviewing process. Such a process indicates the domain knowledge of these reviewers towards the defined problems. The above statements are executed in such a manner that reviewer 1(rev1) marks the article [2] as 1 based on the satisfaction of the quality parameters. The same reviewer (rev1) assesses the quality of the article [55] with parameters of quality assessment; such a reviewing process is applied to all selected articles. Figure 3 highlights the different behaviors of different reviewers towards the selected articles as the process is described in Fig. 4.

## Discussion

Before highlighting the results from the analysis, the bibliometric data and overview of these studies are initially reported. Table 7 highlights the overview of all selected studies and bibliometric information.

**Table 7:** Bibliometric information of selected articles for this SLR

**Identifier**	**Reference**		**Citation count**	**Avg. citations count/year**	**RQ1**	**RQ2**	**RQ3**	**RQ4**
		**Year**						
S1	[3]	2013	41	8.2	✔	X	X	X
S2	[5]	2015	12	2.4	✔	✔	X	X
S3	[10]	2015	2	0.66	✔	✔	X	✔
S4	[11]	2015	9	3	X	X	X	✔
S5	[12]	2017	1	0.5	✔	X	X	✔
S6	[15]	2016	10	2	X	X	X	✔
S7	[22]	2015	1	0.33	X	X	X	✔
S8	[25]	2016	1	0.5	✔	X	X	✔
S9	[28]	2014	0	0	✔	✔	X	✔
S10	[29]	2016	0	0	✔	X	X	✔
S11	[30]	2015	6	2	X	X	X	✔
S12	[32]	2017	0	0	X	X	✔	✔
S13	[36]	2016	7	2.33	X	X	✔	✔
S14	[44]	2016	1	0.5	✔	✔	X	✔
S15	[46]	2016	0	0	✔	✔	X	✔
S16	[56]	2017	1	0.5	X	X	✔	✔
S17	[48]	2015	0	0	✔	✔	X	✔
S18	[49]	2016	0	0	✔	✔	X	X
S19	[50]	2015	12	4	X	X	X	✔
S20	[51]	2010	23	2.8	X	X	✔	X
S21	[52]	2016	7	3.5	X	X	X	✔
S22	[53]	2013	8	1.6	✔	✔	✔	✔
S23	[54]	2017	1	0.5	X	X	✔	✔
S24	[57]	2008	162	18	✔	✔	✔	✔
S25	[58]	2015	5	1.66	X	X	✔	✔
S26	[59]	2017	3	1.5	X	X	✔	X

**Fig. 5:**
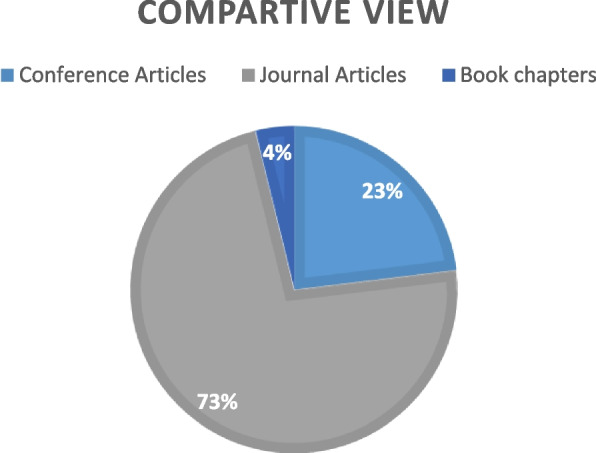
Categorization of selected articles in the form of pie view

The construction of the above table highlights the research questions addressed in different 26 rectified articles. Representation of the ✓ defines the research question address up to satisfied level. Similarly ✗ represents the unsatisfactory results that are not addressed to any research questions. The above construction represents that selected articles are in a range of years 2008 to 2023.However, taxonomical view some artciles consider from 1990 to 2007. Article [57] is the most cited article in the database of 26 research articles. This article addresses the three research questions except research question 3.. Figure 5 display the percentage level of conference, journal and book chapters [37]

## Methodological comparison

**Fig. 6:**
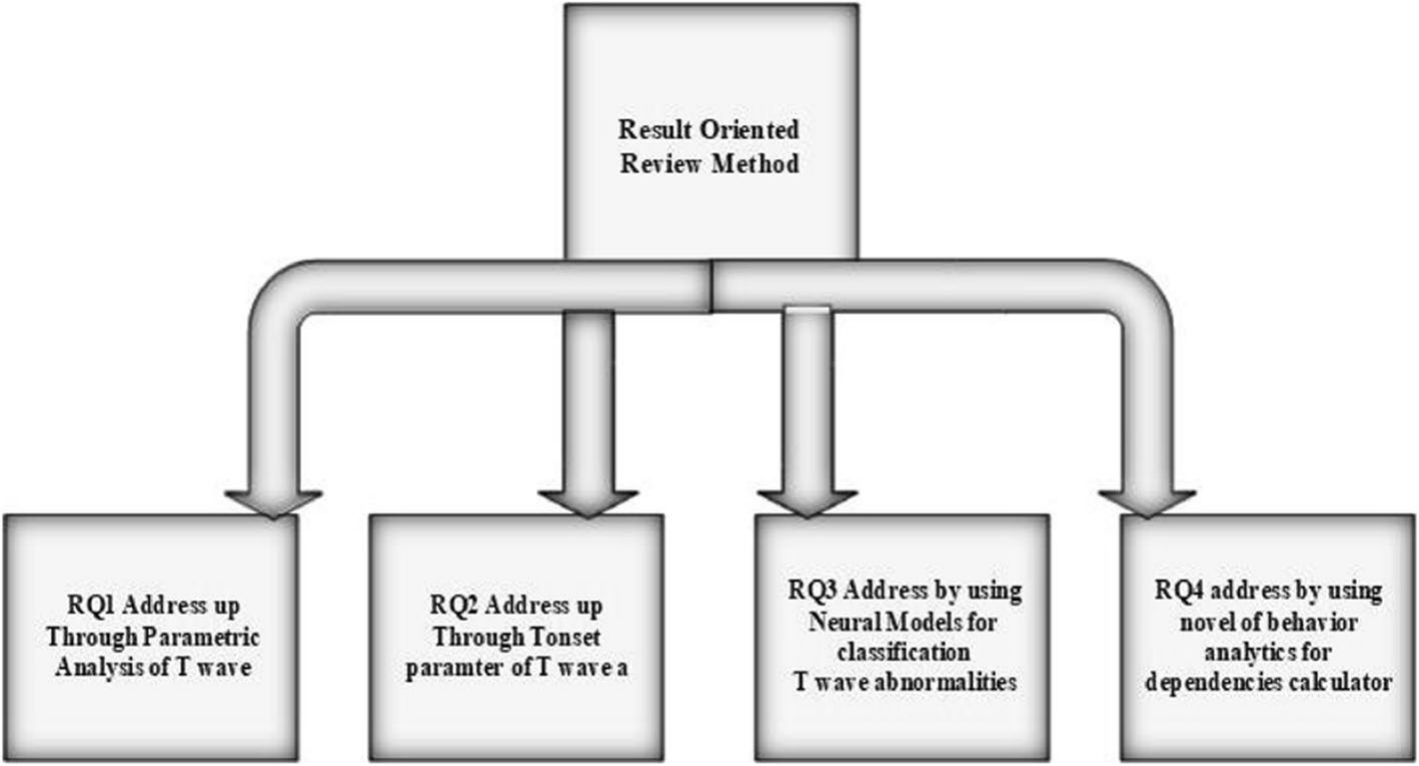
Categorization of research questions address up for result generation

ECG researchers and cardiologists highly value the classification of various T-wave episodes with minimal ambiguity. In the context of this aim, such study is executed in the form of different operational investigations processes that represented the solutions to the defined problems [38, 39]. With the aim of this solutions, an increasing number of studies have found that relates the parametric importance of different ECG features [40]. According to literature, these parametric factors rely on external or internal variables, which can be referred to as feature dependencies [41, 43, 45]. Various state-of-the-art classification techniques for different T-wave patterns are sourced from the existing literature [58, 59]. In the construction of combo pack of solution for better classification of T wave anomalies, firstly designed or identified the best possible research questions after reviewing the literature [5, 50–53]. These research questions are analyzed in a systematic way and then report the solutions to these questions in a combo pack solution (better classification and visibility of T wave anomalies). Figure 6is a showcase categorization of research questions [60, 61].

### RQ1: Parametric analysis of T wave

**Fig. 7:**
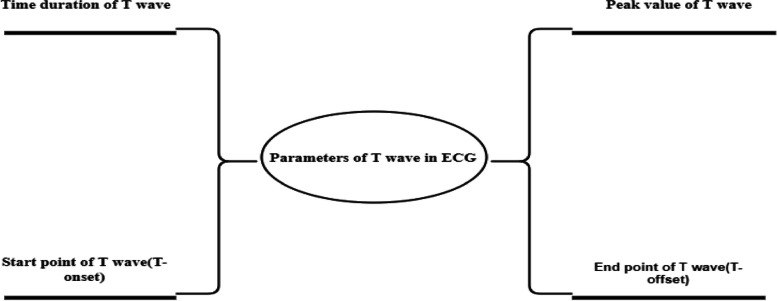
Parametric analysis of T wave in ECG

Distinguishing typical and non-invasive patterns in T-waves is crucial for identifying the nature of myocardial infarction (MI). For achieving such fruitful results of classification by recognizing the keynotes. An increasing number of studies found during the process of literature review that different features of ECG are dependent upon their own parameters. By the help of these parameters’ the possibility of natural extraction of any feature is increased one. The importance of parametric solutions in the case of T wave is always on the high node. Literature displays the four critical parameters of T wave for the extraction of the nature of T wave during the classification. Identification of T wave parameters T-onset, T-offset, T-peak, and T-duration are helpful in own nature extraction and also fruitful for different analysis and techniques like beat-to-beat analysis, the myocardial infarction detection algorithm, and the T-wave alternans detection algorithm (TWA) are discussed in [11, 12, 22]. In beat-to-beat analysis, results are based on the QT interval, which is determined by the parameters Q-onset (start time) and T-offset (end time). Likewise, the myocardial infarction detection algorithm employs the window detector method to capture the peak value of the T-wave (T-peak). Finally, the TWA detection algorithm operates using the R peak as a fiducial point to calculate the T-onset. This algorithm mainly highlights the intensity of the T wave and also reflects the dependencies factor of features that indicate other research problems. [62–65] Such a research problem is a part of this SLR in RQ4. Below Fig. 7 is a showcase of four parameters of T wave [66, 67]

### RQ2: T wave anomalies through a T-onset parameter

Identification of abnormalities in any time series in the dataset is highly desirable especially when you work out on any healthcare system [68–70]. Sometimes these systems deal with a risky situation like a case of MI in ECG. In connection with previous statements, accurate MI identification is essential for diagnostic purposes, as it addresses high-risk factors. In the diagnosis of MI, the primary focus is on recognizing sudden, non-invasive changes in the T-wave; this is a crucial initial step is to highlight the abnormal T-wave episodes [71, 72]. Better classification T wave anomalies depends on a parametric solution but primarily relied on T-onset parameters. An increased number of studies were found during the investigation of dependencies calculation of the T-onset parameter. T wave alterations detection (TWA) algorithm is one of them that is picked from the literature. This algorithm primarily functions to emphasize the energy intensity of the T-wave [73]. The TWA algorithm operates based on the R peak as a fiducial point and takes into account the RR intervals [22, 74]. A key component of the TWA algorithm is the RR interval, which is represented in the equation below. The presentation of this equation reflects the energy intensity of the T-wave, whether in a standard form or as anomalies, depending on the T-onset parameter of the T-wave [22].

$$T_{on} = 40 + 1.33\sqrt {RR}$$

*4.3 RQ3: Classification through Neural Models.* Detection of the irregularity in the sequences of data reveals the difference like the data. The classification process is commonly employed to identify irregular or unconventional patterns (novelty detection) in ECG data. This process differentiates between regular and irregular patterns. Traditionally, various ionic classification techniques are utilized to distinguish irregular responses, except for the predictive element. In cases of rapid and accurate classification commonly used methodologies are a neural network (NN) or artificial neural network (ANN) [75–78]. Figure 6 refers to the basic structure of NN with the inclusion of input, hidden, and output layers for classification.

Conducting operational investigations in any neural network methodology [79–81] involves a structured approach. The primary objective is to train the neural network by adjusting the weights of each unit to minimize the error between the desired output and the actual output. Error derivation of the outputs is a critical phase that shows changes in error as the weights are adjusted slightly, either increased or decreased [58]. The Back Propagation (BP) algorithm is commonly employed to assess and minimize error. The primary focus of this study is to classify anomalies in the T-wave of ECG signals using a feed-forward multi-layer neural network in conjunction with the backpropagation algorithm. In an ideal scenario, the neural network's target values closely approximate the expected output values. The Levenberg–Marquardt algorithm has been found to yield the best results in this context. The primary achievement of this algorithm is the accurate discrimination between two types of heartbeats, namely regular heartbeats and premature ventricular contractions (PVC). [32, 59].

**Fig. 8:**
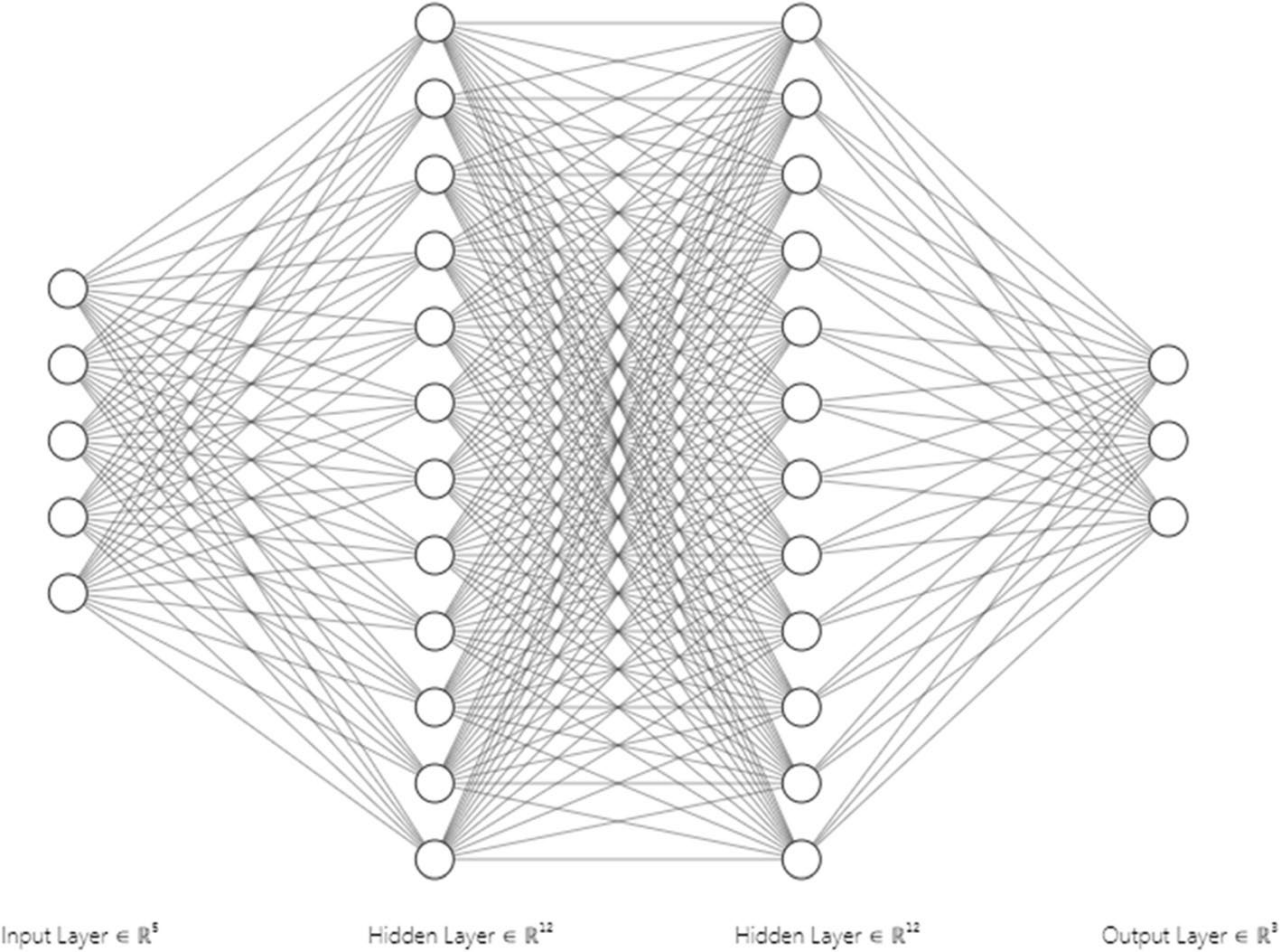
The basic structure of the neural network, in input, hidden, and output layers are essential builder part

Another success story of the artificial neural network (ANN) as in Fig. 8 is to detect the most significant part of the ECG waveform (QRS complex detection). In the scenario of accurate detection of the QRS complex, after the R peak detection by the formation of the feature vectors under usage the amplitude of the significant frequency components of the DFT frequency spectrum [58]. By using the knowledge from the literature, categorized the classification of different features of ECG through two different scenarios. One is a supervised scenario in which training samples are labeled as a standard or anomalous (abnormal) [82, 83]. The second one is unsupervised scenarios; this scenario works in such a manner that training samples are inducted in the neural network without any label [53, 54].

### RQ4: Dependencies impact of the T wave

An anomalous pattern classifier is a valuable tool for cardiac specialists, particularly in high-risk situations such as the sudden onset of myocardial infarction (MI). The initial step in identifying these patterns, especially in the case of MI, is to pinpoint the underlying cause by analyzing T-wave changes [84, 85]. Understanding the root cause becomes feasible by highlighting the dependencies of the T-wave. Calculating these dependencies requires having access to all the parameters of the T-wave, including T-offset, T-wave duration, T-wave amplitude, and T-onset values, which are essential for accurately tagging T-wave anomalies All these parameters have equal importance that already discussed in the above address up part of RQ1, but T onset parameter has a unique concentration in case of extraction the nature of the anomalies. In the literature review session, Numerous studies have contributed to ECG feature analysis, and this analysis is often conducted using the advanced methodology known as Wavelet Transform Module Maxima (WTMM). The T-wave detection algorithm employs WTMM to identify T-waves, relying on various T-wave parameters, including a combination of T-wave amplitude and slope. In addition to this, another state-of-the-art approach, the Trapezium method, is utilized for T-wave detection, primarily through the T-peak amplitude parameter. These two state-of-the-art methods also indirectly aid in uncovering dependencies. In connection with such a statement, the same case is applied to different ECG features by using the above concept of dependencies. Highlighting the dependencies factors are quite hard without finding the exact nature of any ECG feature [86–88]. Similarly, in the specific case of two T-wave anomalies, T-wave inversion, and flattened T-waves, they are often grouped together, yet their behaviors in the context of MI differ significantly. To emphasize the distinctions between inverted and flattened T wave, we need some sort of novelty in our solution [89, 90]. For the achievement of this aim, we need particular checkpoints that are worked on the basis of the parametric solution, like behaviors identifier that worked in the Ontology behavior model (OntoB). The ontology behavior model is a composition of several factors which are briefly discussed in below Fig. 13 and the flow of these factors is highlighted [15, 30, 52].

**Fig. 9:**
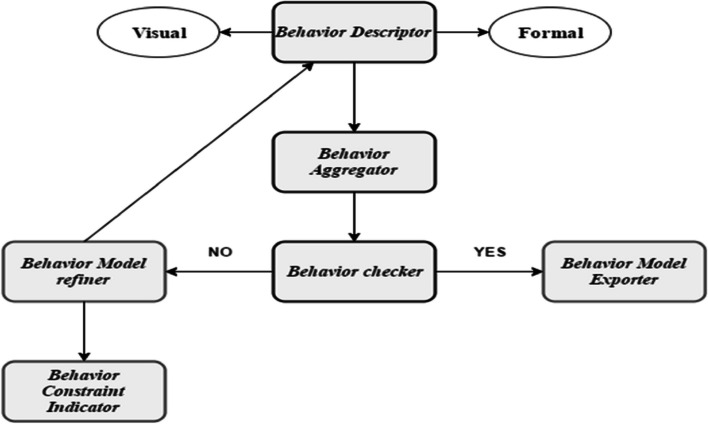
Ontology Behavior model: Identification of intercouple and intracouple relationship in behaviors and behavior refiner

Behavior descriptor: Behavior descriptor represents the core behavior properties or elements. Behavior Aggregation: Combination of behaviors hierarchical and hybrid These components are integrated to produce Semitics of TS through both intra-coupling and inter-coupling relationships within a set of behaviors [56]. The Behavior Constraint Indicator module formalizes natural language descriptions of behaviors into logical formulas. The Behavior Checker module serves as a checkpoint to verify the accuracy of the behavior constraints and properties claimed by the Behavior Constraint Indicator [51]. The Behavior Model Refiner module is used to make further refinements to the behavior model, addressing any identified issues. Finally, the Behavior Model Exporter exports a stable and desired behavior model as a result of the modeling process [91, 92]. Figure 9 higlighted the ontology behavior model.

Efficient formation of the classifier is highly desirable for tagging the anomalies in ECG signals due to life care issues. We cannot afford any minor ambiguity in the monitoring process of the heart. ECG signals would be real-time patient’s heart data and risky if we missed any activity at any particular time that may cause the death of the patient. For the last decade, cardiologists and medical professionals are still searching for a way to highlight the intensity of myocardial infarction. This intensity is measured with the help of dependencies factors. In one scenario these dependent factors are measured with the help of changes in the T wave or anomalies of T wave [48]. For reduction of risky situations by integration the novel approach for determining the dependencies which are helpful for broad classification of different T wave episodes. Analysis of ECG is primarily dependent upon the classification result which represents the regularity and irregularity in ECG signals [29].

**Fig. 10:**
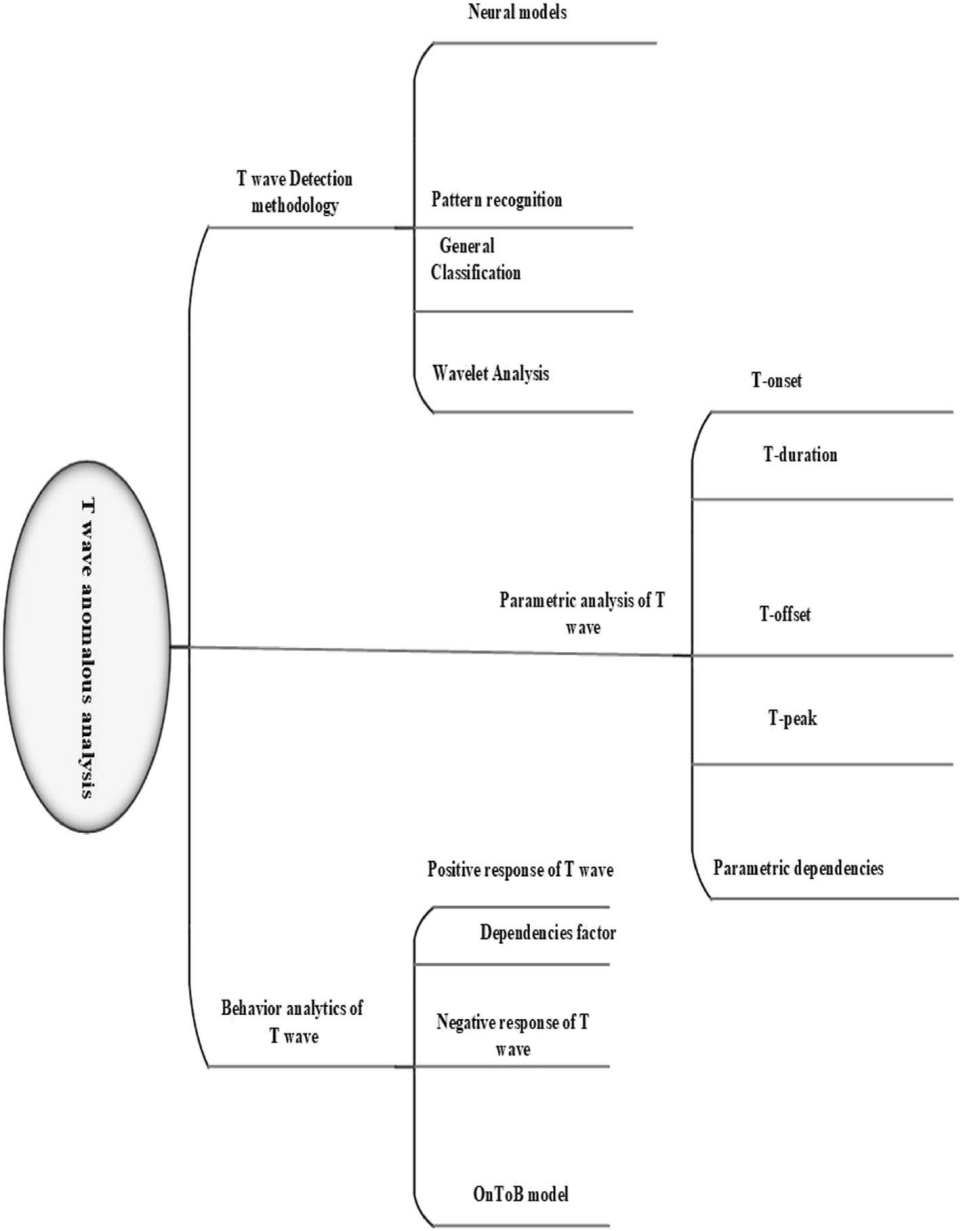
Classification of T wave anomalies analysis

Considering the focus of this article, our primary concern is to find a way how it is possible to get robust and accurate results of diverse T wave classification. In the literature survey process, a lot of research found that worked in the form of, different methodologies and techniques for T wave classification. But efficient and accurate results have always attracted the researchers. In-depth analysis of literature, survey process creates a path for solution-oriented results. This idea works on the basis of three different perspectives, such are T wave parametric analysis, the combination of feature analysis of ECG and the behavior analytics techniques in the light of coupling concept of behavior analytics quire clears the concept of dependencies of T wave, finally add up the backpropagation and multi feed-forward algorithms of neural network in different models of neural network. Figure 14 is the pictorial representation of our proposed idea towards the robust and accurate classification of different T wave episodes. Figure 10 represnts the parametric classfication of different T-Wave anomalies.

The most important part of this proposed study is our critical analysis that we get the knowledge after the literature survey session. According to our knowledge so far, we are the first ones who discussed the dependencies factor for the robotic classification of T wave anomalies. Such discussion will not be limited to robotic classification of T wave anomalies, but also creates a new room for researchers to crucially think about the different features of ECG in the light of dependencies factor. The inclusion of dependencies factor in feature analysis of ECG makes sense for depth analysis of different CVDs like myocardial infarction (MI), atrial fibrillation (AF), and premature ventricular contraction (PVC). Detection of these CVDs depends upon the different features of ECG [93, 94].

## Taxonomical View:Three-Way Handshake

**Fig. 11:**
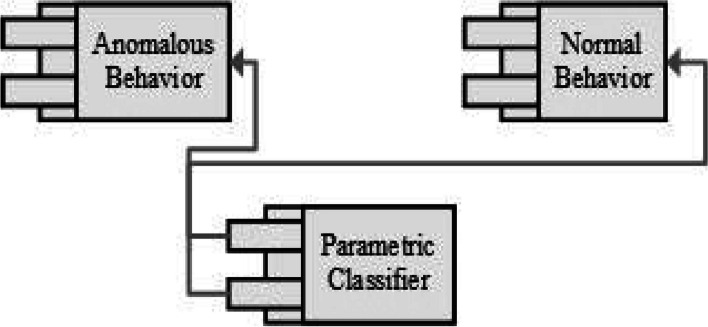
Parametric Classification for T wave

A joint venture of backpropagation approach of neural network (NN), parametric analysis of T wave, and novelty of behavior analytics plays an important role in the rapid and exact classification of T wave anomalies based on nature. Results of the above combination are already achieved as work on singleton, but our approach towards classification is robust and accurate. The methods of DWT for T wave detection, T wave detection algorithm, TRA approach, and wavelet analysis all are worked under the coverage of parametric analysis of T wave classification. Figure 11 indicates the addressing literature in a pictorial model [3, 49, 50].

**Fig. 12:**
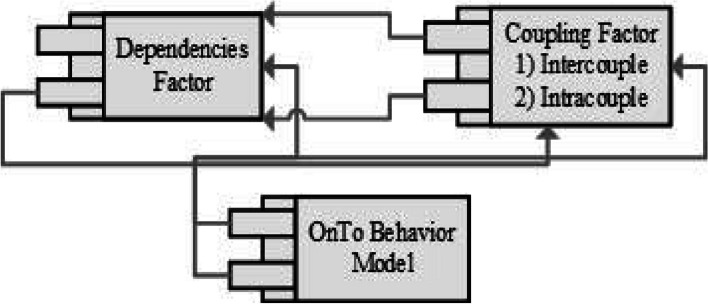
Dependencies perspective of the T wave

The novelty of behavior analytics in this combination is playing the critical role for highlighting the dependences of T wave on the other features of ECG. In latest TWA algorithm, the same concept of features dependencies is reflected in the form of highlighting the T wave changes with the integration of R peak. The dependencies concept is further explained in behavior analytics in the reflection of the intercouple concept of behavioral theory. Below Fig. 12 highlights the concepts of behavior analytics with studied articles [50, 52].

**Fig. 13:**
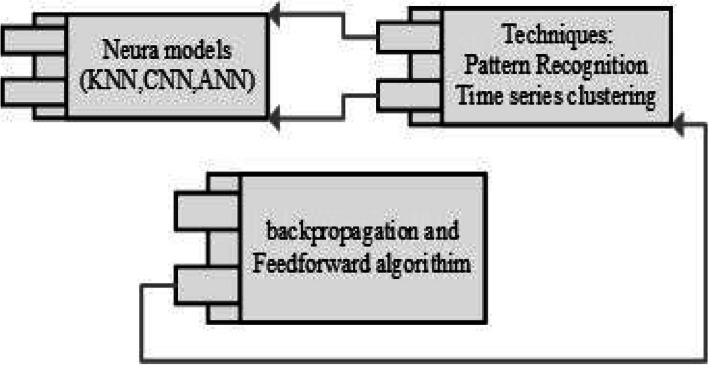
Classification of T wave with NN

In the perspective of a neural network using the combination of feedforward and backpropagation algorithms with the conventional techniques of neural network (pattern recognition and time clustering). Classification of T wave through neural models for the identification of dependencies is clearly highlighted. These dependencies are identified by some checkpoint; Fig. 13 capture the whole scenario [53, 54, 59].

**Fig. 14:**
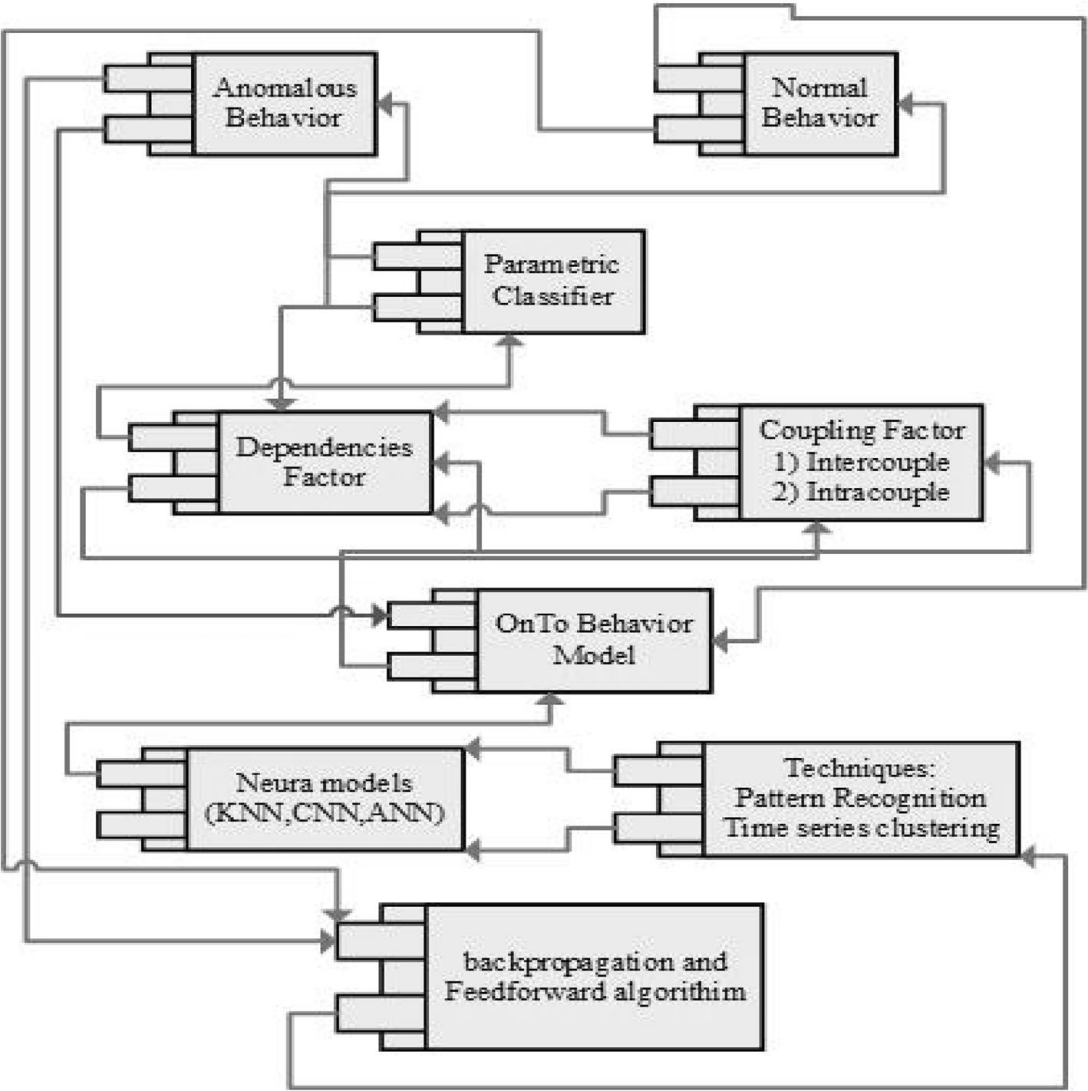
Three-way handshake structure for robust classification of different T wave episodes

A complete package of three-way handshake process is highlighted in Fig. 14. The result of this combination is worked in such manner that the usage of different neural models in the first perspective is for validation and testing of our different results which we get from other two perspectives. In second dependences perspective, the OnToB model is worked on the basis of the output of neural models in a queued manner (first come first serve). This OnToB checks the indication of T wave in the light of coupling concepts of behavior analytics (intercouple and intracouple) that reflects the identification of dependencies factor [95]. This dependencies factor works as an input of the parametric perspective of T wave for the classification of the regular and anomalous T wave [96, 97]. One output of each regular and anomalous T wave is rebooted into the OnToB model and the second output of each regular and anomalous T wave are also rebooted in the combination of algorithms for further subclassification.

## Future research directions

In an ideal case highlighting the CVDs at early time delivers the in time treatment to patients. In Process of extraction of different features in ECG, at first, stage implemented the different noise filtration methods by setting the threshold value. From literature, we got different methodologies for feature extraction with different techniques. But after reviewing the articles, one issue is prevalent in every technique such issue is noise filtration. Reason for such issue is due to lack standard or state-of-the-art work on the threshold value. In case of T wave applied noise filtration, we need some sort of standard baseline value or threshold value (need a peak point and time duration of T wave) like the representation of Table 7 and reading parameters of ECG from Table 8.

**Table 8:** Standard readings of parameters of ECG [57]

Peak Values Amplitude		Intervals Duration	
P-Wave	0.75 mV	P_R Interval	0.12 to 0.20 Sec
R-Wave	1.60 mV	QT Interval	0.35 to 0.49 Sec
Q-Wave	25% of R-Wave	ST Segment	0.05 to 0.15 Sec
T-Wave	0.1 to 0.5 mV	P wave Interval	0.11 Sec
		QRS Complex	0.09 Sec
		PR Segment	0.06 to 0.15 Sec
		T wave	Varies

**Fig. 15:**
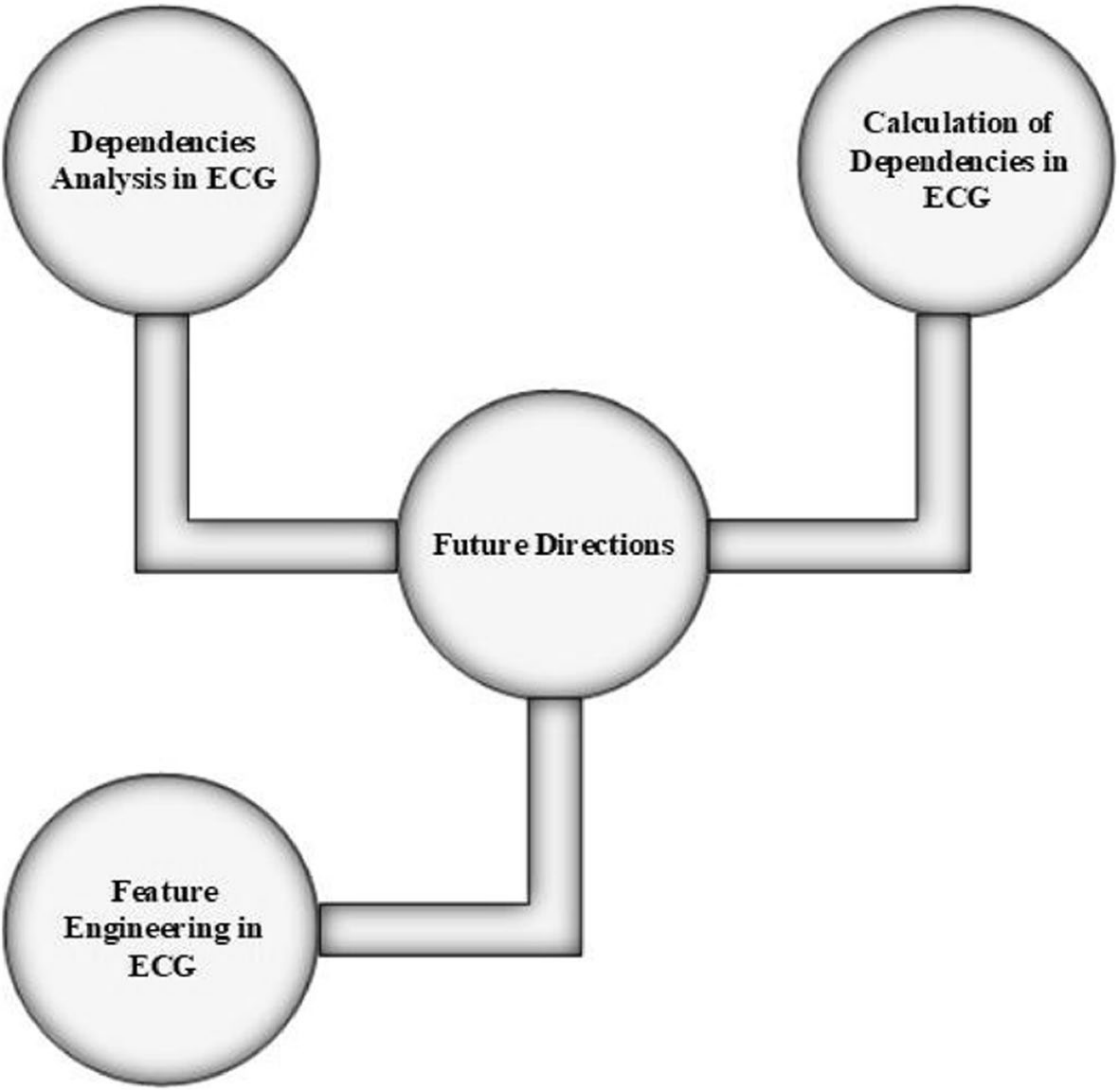
Future direction for ECG analysis in terms of different anomalies analysis

Some essential research directions are highlighted in Fig. 15 after analyzing the selected studies.

1. Analysis of dependencies factor of different CVDs, such analysis helps to provide the predictive information
2. Calculation of dependencies factor through dimensional reduction techniques PCA, SAX on ECG signal
3. Feature engineering embeds on ECG and works with the presence of noise in signal

### Dependency analysis of different CVDs

Identification, the dependencies of each feature, is an ideal case for diagnostic purposes refering to Fig. 15. Such scenario will be implemented through the parameters of each feature as the above parametric discussion of T wave. Every CVDs depends on different features, and such features have some critical parameters. Highlight the dependencies between these parameters; such process will be performed by conducting the parametric analysis along with the coupling concepts of behavior analytics.

### Calculation of dependencies through dimension reduction

Complexities reduction is the best way to proceed the data analysis in smooth and accurate manner. With concern of this ECG analysis, the dimensional reduction is highly demandable due to such reduction calculation of dependencies between different features will be simpler one. Such statement operates in ECG analysis by using the primary tacts of dimensional reduction. Most important part of the ECG is considered QRS complex. Reduction or segmentation of such part will be valuable for better analysis of R peak detection. PAC and SAX techniques will be used for segmentation of QRS complex and also cross-check the results of each technique. With the help of such segmentation, premature ventricular contraction (PVC) detection will be a more accurate job than previous work.

### Feature engineering in ECG

Implementation of feature engineering reduces the noise involvement in ECG signal. In simple words, we will perform better ECG analysis with the presence of noise by embedding the feature engineering. In this engineering components of the system will be an enhancement and scalable for in-depth investigations.

## Conclusion

Rapid and accurate classification of different T wave episodes is highly desirable in terms of identification of T wave anomalies. T wave anomalies is a crucial concern for the ECG researchers in the sense of analyzing the MI. This systematic study highlights the critical question which relates the behavior of T wave anomalies and dependencies between them. The purpose of this article is to find the solution of queries. Proceed this aim by a collection of 97 articles from four prime databases. In next phase set the rectification process for Pursuing a resolution for the specified issue by applying the filtration on 97 articles. Such filtration criteria set Based on the criteria for inclusion and exclusion, literature quality assessment and data extraction strategy. After these stages finally get the 26 refined articles that are the part of this systematic study. These articles included the parametric analysis of T wave, features analysis of ECG for understanding the dependencies of T wave parameters along with articles of behavior analytics for understanding the dependencies factors.

Few gaps are highlighted after the literature survey process, these gaps are related to the requirement of further qualitative improvement of noise filtration technique and feature detector window method. In the end portion of this study, we have used our best knowledge and skills by critically brainstorming activities. Such activities are worked under the coverage of selected articles for the best possible solution for rapid classification of T wave episodes. Our research is based on the joint version of three-way perspective. The proposed method is a three-way handshake that works by inclusion the neural models with their prime algorithms, and after then OnToB behavior model works under the light of coupling concepts of behavior analytics along with parameters of T wave. Such approach is a novelty theme-based solution and a perfect one for the rapid classification of the different T wave episodes.

### Supplementary Information

Supplementary Material 1.

## Data Availability

The extracted process of articles is mainly relying on four primary databases, and these are all available publicly. http://ieeexplore.ieee.org http://link.springer.com http://www.sciencedirect.com https://www.scopus.com

## Footnotes

1 https://www.nih.gov/health-information/nih-clinical-research-trials-you/guiding-principles-ethical-research

2 https://ease.org.uk/communities/peer-review-committee/peer-review-toolkit/how-to-select-reviewers/
