# Supplementary material for: Methodological identification of anomalies episodes in ECG streams: a systematic mapping study
Source: BMC Med Res Methodol. 2024 Jun 4;24:127. doi: 10.1186/s12874-024-02251-0 (PMC11149236; doi:10.1186/s12874-024-02251-0)
Supplement: Supplementary file 1 — Supplementary Material 1. [file 12874_2024_2251_MOESM1_ESM.docx]

**APPENDIX-A**

| **Data Source** | **Data Unit information** |
| --- | --- |
| *Identifier* | S3 |
| *Title* | An ECG T-wave Incongruities Detection Using a Lightweight Classification Model for Wireless Body Sensors |
| *Author* | Medina Hadjem , Farid Naït-Abdesselam |
| *Year of Publication* | 2015 |
| *Publication Type ( Journal /Conference)* | Conference |
| *Publication Venue* | Workshop on ICT-enabled services and technologies for eHealth and Ambient Assisted Living, IEEE ICC. |
| *Research Method ( Experimental Approach/ SLR / A case study)* | T wave detection through WBS experimentall investigations |
| *Research Questions Address up* | *Directly: RQ1 , RQ2, and RQ4 satisfied* |
|  | *Indirectly*: ------------ |
|  |  |
| **Data Source** | **Data Unit information** |
| *Identifier* | S4 |
| *Title* | Robust and Accurate Anomaly Detection in ECG Artifacts Using Time Series Motif Discovery |
| *Author* | Haemwaan Sivaraks . Chotirat Ann Ratanamahatana |
| *Year of Publication* | 2015 |
| *Publication Type ( Journal /Conference)* | Journal |
| *Publication Venue* | Computational and Mathematical Methods in Medicine |
| *Research Method ( Experimental Approach/ SLR / A case study)* | ECG feature analysis through experimental investigations |
| *Research Questions Address up* | *Directly: ------------* |
|  | *Indirectly*: RQ4 satisfied |
|  |  |
| **Data Source** | **Data Unit information** |
| *Identifier* | S5 |
| *Title* | Benchmarking of T wave Alternans detection method on empirical mode decomposition |
| *Author* | Manuel Blanco–Velascoa,_, Rebeca Goya–Estebanb, Fernando Cruz–Roldána, Arcadi García–Alberolac, José Luis Rojo–Álvarezb |
| *Year of Publication* | 2017 |
| *Publication Type ( Journal /Conference)* | Journal |
| *Publication Venue* | Computer Methods and Programs in Biomedicine |
| *Research Method ( Experimental Approach/ SLR / A case study)* | TWA detection on the basis of hypotheses( Experimental investigations) |
| *Research Questions Address up* | *Directly: RQ1, RQ4 satisfied* |
|  | *Indirectly*: ------------------------- |
|  |  |
| **Data Source** | **Data Unit information** |
| *Identifier* | S6 |
| *Title* | Combined Mining: Analyzing Object and Pattern Relations for Discovering and Constructing Complex Yet Actionable Patterns |
| *Author* | Longbing. Cao |
| *Year of Publication* | 2013 |
| *Publication Type ( Journal /Conference)* | Journal |
| *Publication Venue* | Wiley Interdisciplinary Rev.: Data Mining and Knowledge Discovery |
| *Research Method ( Experimental Approach/ SLR / A case study)* | Behavior Analytics of real system through review study |
| *Research Questions Address up* | *Directly: ------------* |
|  | *Indirectly*: RQ4 satisfied |
|  |  |
| **Data Source** | **Data Unit information** |
| *Identifier* | S7 |
| *Title* | A combined algorithm for T-wave alternans qualitative detection and Quantitative Measurement |
| *Author* | Kelvin KF Tsoi, Yong-Hong Kuo, Helen M. Meng |
| *Year of Publication* | 2013 |
| *Publication Type ( Journal /Conference)* | journal |
| *Publication Venue* | Journal of Cardiothoracic Surgery |
| *Research Method ( Experimental Approach/ SLR / A case study)* | Deterministic approach for finding T-onset through experimental work |
| *Research Questions Address up* | *Directly:* RQ1,RQ2 satisfied |
|  | *Indirectly*: RQ4 satisfied |

**APPENDIX-B**

| **ARTICLE SCORING CALCULATOR** | | | | | | | |
| --- | --- | --- | --- | --- | --- | --- | --- |
| **Reference** | **Paper Title** | **Rev 1** | **Rev 2** | **Rev 3** | **Rev 4** | **Rev 5** | **Total Score** |
| [2] | ECG signal processing for abnormalities detection using  multi-resolution wavelet transform and Artificial Neural Network classifier | 1 | 0.5 | 0 | 0 | 1 | 2.5 |
| [3] | ECG Patch Monitors for Assessment of Cardiac Rhythm  Abnormalities | 1 | 0.5 | 0 | 0.5 | 1 | 3 |
| [4] | Electrocardiography-inclusive screening strategies for  detection of cardiovascular abnormalities in high school athletes | 0.5 | 0.5 | 0.5 | 0 | 0.5 | 2 |
| [7] | A Novel Multi-Resolution SVM (MR-SVM) Algorithm to  Detect ECG Signal Anomaly in WE-CARE Project | 1 | 0.5 | 0 | 1 | 1 | 3.5 |
| [11] | Heartsaver: A mobile cardiac monitoring system for  auto-detection of atrial fibrillation, myocardial infarction,  and atrio-ventricular block | 1 | 0 | 0 | 0.5 | 0 | 1.5 |
| [12] | An ECG T-wave anomalies Detection Using a Lightweight  Classification Model for Wireless Body Sensors | 1 | 0 | 1 | 0.5 | 0.5 | 3 |
| [13] | Robust and Accurate Anomaly Detection in ECG Artifacts  Using Time Series Motif Discovery | 0.5 | 1 | 0.5 | 1 | 0.5 | 3.5 |
| [14] | Benckmarking of T wave Alternans detection method on empirical mode decomposition | 1 | 0.5 | 0 | 1 | 1 | 3.5 |
| [17] | Combined Mining: Analyzing Object and Pattern Relations for Discovering  and Constructing Complex Yet Actionable Patterns, | 0 | 1 | 1 | 0 | 1 | 3 |
| [24] | A combined algorithm for T-wave alternans qualitative detection and quantitative Measurement | 1 | 1 | 0 | 0 | 1 | 3 |
| [27] | Improved Abnormality Detection from Raw ECG Signals  using Feature Enhancement | 1 | 0.5 | 1 | 0.5 | 0 | 3 |
| [28] | Risk prediction for cardiovascular disease using ECG data in  the China kadoorie biobank | 1 | 0 | 1 | 0 | 0 | 2 |
| [30] | ECG Feature Extraction in Temporal Domain and Detection  of Various Heart Conditions | 1 | 1 | 1 | 0 | 1 | 4 |
| [31] | Simple T wave metrics may better predict early ischemia  as compared to ST segment | 1 | 1 | 1 | 0.5 | 1 | 4.5 |
| [32] | Formalization, and Verification of Group Behavior Interactions | 1 | 0 | 1 | 0 | 1 | 3 |
| [33] | Non-IIDness Learning in Behavioral and Social Data | 0 | 0 | 0 | 0 | 0.5 | 0.5 |
| [34] | Cardiologist-Level Arrhythmia Detection with Convolutional Neural Networks | 1 | 0.5 | 0.5 | 0.5 | 1 | 3.5 |
| [35] | Diagnosis of Cardiovascular Abnormalities From  Compressed ECG: A Data Mining-Based Approach | 0 | 0.5 | 0 | 1 | 0 | 1.5 |
| [38] | Neighborhood rough set based ECG signal classification | 1 | 0.5 | 1 | 0.5 | 1 | 4.5 |
| [39] | Identifying Usage anomalies For ECG-based Sensor Nodes | 0 | 0.5 | 0 | 0 | 0.5 | 1 |
| [40] | Real-Time ECG Signal Feature Extraction for the Proposition  of Abnormal Beat Detection – Periodical Signal Extraction | 0.5 | 0.5 | 0.5 | 0 | 0.5 | 2 |
| [41] | Design of a Real-time Morphology-based Anomaly Detection  Method from ECG Streams | 0.5 | 0.5 | 0 | 0.5 | 0.5 | 2 |
| [46] | ST segment/heart rate hysteresis improves the diagnostic  the accuracy of ECG stress test for coronary artery disease in  patients with left ventricular hypertrophy | 1 | 1 | 0 | 1 | 1 | 4 |
| [48] | Statistical approach for lightweight detection of anomalies  in ECG | 1 | 0.5 | 1 | 0.5 | 1 | 4 |
| [49] | Web-based and mobile system for training and improving  in the field of electrocardiogram (ECG) | 0 | 0 | 0 | 0 | 0 | 0 |
| [51] | Minimal T-wave representation and its use in the assessment of drug arrhythmogenicity | 1 | 1 | 0.5 | 0.5 | 0.5 | 3.5 |
| [55] | A new method for removal of power line interference in  ECG and EEG recordings | 0.5 | 0.5 | 1 | 0.5 | 0.5 | 3 |
| [59] | ST-segment and T-wave anamalies Prediction in an  ECG Data Using RUSBoost | 1 | 0.5 | 0 | 0.5 | 1 | 3.5 |
| [60] | Identifying usage incongruities for ECG-based sensor nodes | 0 | 1 | 0 | 1 | 0 | 2 |
| [62] | A woman with recurrent chest pain and ST-segment elevation | 0 | 0 | 0 | 0 | 0 | 0 |
| [66] | Telecardiology: Hurst Exponent based Anomaly Detection  in Compressively Sampled ECG Signals | 0.5 | 0.5 | 0 | 0.5 | 0.5 | 2 |
| [76] | Ground Delay Program Analytics with Behavioral Cloning  and Inverse Reinforcement Learning | 0 | 0 | 0 | 0 | 0.5 | 0.5 |
| [78] | An Efficient GA-Based Algorithm for Mining Negative  Sequential Patterns | 1 | 0.5 | 1 | 0 | 1 | 3.5 |
| [80] | Mining Both Positive and Negative Impact-Oriented  Sequential Rules from Transactional Data | 0 | 0 | 0.5 | 0.5 | 0.5 | 1.5 |
| [81] | e-NSP: Efficient Negative Sequential Pattern Mining | 0.5 | 1 | 0.5 | 0.5 | 1 | 3.5 |
| [82] | Artificial Neural Network-Based Automated ECG Signal  Classifier | 0.5 | 0.5 | 1 | 0.5 | 0.5 | 3 |
| [83] | Basic concepts of artificial neural network (ANN) modeling  and its application in pharmaceutical research | 0 | 0 | 0 | 0 | 0 | 0 |
| [84] | Pattern recognition techniques for automatic detection of  suspicious-looking incongruities inmammograms | 1 | 1 | 0 | 1 | 1 | 1.5 |
| [87] | Patient-specific deep architectural model for ECG classification | 0.5 | 0.5 | 1 | 1 | 1 | 4 |
| [88] | ECG Signal Analysis Using Wavelet Transforms | 0.5 | 1 | 0.5 | 1 | 1 | 4 |
| [89] | “ Heart Sound Anomaly and Quality Detection using  Ensemble of Neural Networks without Segmentation | 0.5 | 1 | 0.5 | 0.5 | 0.5 | 3 |
| [92] | Convolutional Neural Networks for Patient-Specific ECG  Classification | 0.5 | 0 | 0 | 0 | 0.5 | 1 |
| [93] | ECG-Based Classification of Resuscitation Cardiac Rhythms for Retrospective Data Analysis | 1 | 0.5 | 0 | 0.5 | 1 | 3 |
|  |  |  |  |  |  |  |  |
